# Sustainable Biopolymer Films from Amazonian Tambatinga Fish Waste: Gelatin Extraction and Performance for Food Packaging Applications

**DOI:** 10.3390/foods14223866

**Published:** 2025-11-12

**Authors:** Fernanda Ramalho Procopio, Rodrigo Vinícius Lourenço, Ana Mônica Q. B. Bitante, Paulo José do Amaral Sobral, Manuel Antônio Chagas Jacintho

**Affiliations:** 1 Embrapa Pecuária Sudeste, São Carlos 13560-970, SP, Brazil; manuel.jacintho@embrapa.br; 2 Department of Food Engineering, Faculty of Animal Science and Food Engineering, University of São Paulo, Av Duque de Caxias Norte, 225, Pirassununga 13635-900, SP, Brazil; rodrigolourenco@usp.br (R.V.L.); amonica@usp.br (A.M.Q.B.B.); 3 Food Research Center (FoRC), University of São Paulo, Rua do Lago, 250, Semi-Industrial Building, Block C, São Paulo 05508-080, SP, Brazil

**Keywords:** fish processing, fish by-product, protein, edible films, sustainable development

## Abstract

Tambatinga (*Colossoma macropomum* × *Piaractus brachypomus*), a hybrid Amazonian fish recognized for its superior growth performance, represents a valuable and sustainable source of collagen-rich raw material. Due to its tropical origin, the species’ skin may contain higher levels of amino acids, which can enhance the functional and structural properties of gelatin derived from it. The valorization of fish processing residues for biopolymer production not only mitigates environmental impacts but also reinforces the principles of the circular economy within aquaculture systems. This study explores the development of biopolymer films from Tambatinga skin, an abundant by-product of Brazilian aquaculture. The skins were cleaned and subjected to a hot water–acid extraction process to obtain gelatin. The extracted gelatin exhibited high proline and hydroxyproline contents (12.47 and 9.84 g/100 g of amino acids, respectively) and a Bloom strength of 263.9 g, confirming its suitability for film formation. Films were prepared using 2 g of gelatin per 100 g of film-forming solution, with glycerol added at 10 and 20 g/100 g of gelatin. The resulting films were transparent, flexible, and showed uniform surfaces. Increasing the glycerol concentration reduced tensile strength (from 59.4 to 37.9 MPa) but improved elongation at break (from 116% to 159.1%) and modified the films’ thermal behavior. Moreover, Tambatinga gelatin films demonstrated excellent UV-blocking performance (below 300 nm) and lower water vapor permeability compared to other gelatin-based films reported in the literature. These findings highlight the potential of fish skin—typically regarded as industrial waste—as a renewable and high-value raw material for the production of sustainable biopolymers. This approach supports resource efficiency, waste reduction, and the broader goals of sustainable development and circular bioeconomy.

## 1. Introduction

The fish industry represents one of the fastest-growing segments of the global food sector. According to the Food and Agricultural Organization Yearbook of Fishery and Aquaculture Statistics [1], the worldwide production of fish, crustaceans, and mollusks reached 177.8 million tons in 2019, with approximately 89% of total aquaculture and fisheries output destined for direct human consumption. China, India, Indonesia, Vietnam, and Bangladesh rank among the world’s leading producers. In many developing and underdeveloped nations, strengthening the fish production chain can play a crucial role in ensuring the availability of affordable, high-quality animal protein [2]. Although Brazil is not yet among the major global producers, the country holds considerable potential for expansion due to its abundant water resources and the diversity of its native species.

Brazilian fish production has increased by 45% since 2014, being Tambaqui (*Colossoma macropomum*) standing out as the leading native species [3]. Tambatinga (*C. macropomum* × *Piaractus brachypomus*, *Characidae*), obtained by crossing a Tambaqui female with a Piraptinga (*P. brachypomus*) male, is commonly cultivated in fish farming tanks due to its broader commercial availability. Hybrid cultivation is generally preferred because of the improved productive performance of these species. Generally, hybrid cultivation is preferred due to the better productive performance. Specifically, Tambatinga is valued by producers for its rapid growth rate, hardiness, and high carcass yield [4]. Within national aquaculture production, the group of hybrids—Tambacu (female *C. macropomum* × male *Piaractus mesopotamicus*, known as Pacu) and Tambatinga—ranks fourth in production volume, underscoring their significant role in the advancement of Brazilian aquaculture.

One of the major challenges in the fish production chain is the substantial amount of waste generated during processing. Skins, heads, scales, viscera, and bones can represent up to 50% of the animal’s total weight [5]. Although these residues are commonly used in the production of animal feed, certain fractions—particularly fish skin—represent a valuable source of collagen and bioactive peptides. These components have attracted increasing attention for their potential applications in the development of biodegradable films, scaffolds, and functional food supplements [6,7].

Collagen is the most abundant animal protein, constituting connective tissues, skin, ligaments, cartilage, and tendons, formed by three polypeptide chains structured as a triple helix [8]. The chains are formed by sequential units of Glycine-X-Y, with the X and Y positions occupied mainly by proline and hydroxyproline [9]. Approximately 28 types of collagens have been identified, with type I collagen being the most common [10]. Its primary function in bodies is to provide structural support and mechanical functionality.

The conversion of collagen to gelatin takes place through partial hydrolysis of the protein structure. Usually, alkaline or acid pre-treatment followed by thermal denaturation in water is used. In the case of peptides, the chains are cleaved into small fragments by enzymatic action. The health benefits linked to collagen, gelatin, and peptides have driven market growth in recent years. According to a study by Grand View Research [11], the gelatin market reached USD 5.80 billion in 2021 and is expected to grow at an annual rate (CAGR) of 9.5% until 2030. The application of collagen and its derivatives ranges from medical and pharmaceutical sectors (tissue regeneration, artificial skin manufacturing, and bone grafting) to the food industry, as a source of peptides, emulsifying agent, and film former [9].

Producing films from agro-industrial waste is a trend that contributes to reducing the environmental impact, corresponding to a Circular Economy approach [12,13]. Moreover, their incorporation into biopolymers, food packaging, pharmaceuticals, and biomedical products adds economic value to the aquaculture sector while fostering an integrated and environmentally responsible production chain. Many authors have demonstrated methods of recovering waste from aquatic sources to produce films with good optical and mechanical properties [14,15,16,17]. Apart from packaging applications, fish gelatin has also emerged as an interesting source for collagen derivatives since it has greater acceptance among consumers of the most diverse cultures and religions. However, fish collagen may have inferior technological properties compared to mammalian sources. The main reason is the lower concentration of proline and hydroxyproline amino acids [18]. According to Muyonga et al. [19], while mammalian gelatin presents approximately 30% of proline and hydroxyproline content, cold-water (Cod) and warm-water (Tilapia and Nile perch) fish gelatin present 17 and 22–25%, respectively.

Considering the better characteristics of collagen obtained from warm-water sources [20,21], exploring the aspects of Tambatinga skin as a source of gelatin in film production may be of potential scientific and technological interest. Therefore, this work aimed to produce gelatin from Tambatinga skin, evaluating its characteristics as a raw material for film production.

## 2. Materials and Methods

### 2.1. Materials

Frozen Tambatinga fish (*Colossoma macropomum* × *Piaractus brachypomus)* were purchased from Pescados do Vale (Ariquemes, RO, Brazil), distributed by Mar e Rio Pescados (São José do Rio Preto, SP, Brazil). All the reagents used were of analytical grade.

### 2.2. Methods

#### 2.2.1. Separation and Cleaning of Tambatinga Skins

The fish were manually filleted, simulating industrial processing. The separated skins were cleaned to remove meat and fat residues, salted, vacuum-packed, and stored under refrigeration until the day of processing.

#### 2.2.2. Tambatinga Skin Composition

Moisture, ash, and the crude protein content (Kjeldahl) of Tambatinga skin were determined according to the Brazilian Compendium of Animal Feed standard methods [22] using a nitrogen conversion factor of 5.62 [23,24]. The fat content was determined according to the standard method [25]. The analyses were performed in triplicate.

#### 2.2.3. Gelatin Production

The Tambatinga gelatin was produced using the methodology detailed in Procopio et al. [26]. The skins were immersed in a 0.1 M sodium hydroxide (NaOH) solution at a 1:8 ratio (skin/solution) and slowly agitated for 2 h to effectively remove non-collagenous globular proteins and residual fat. After washing, the skins were subjected to acid treatment (0.1 M acetic acid, 1:8 ratio) inside a jacketed reactor (Usinox, Batatais, SP, Brazil) under gentle agitation for 16 h. The primary extraction of gelatin was conducted immediately after the acid treatment in the same reactor. The solution was heated to approximately 60 °C and maintained under gentle stirring for 5 h. The resulting hot gelatin solution was subsequently pumped through a preheated stainless steel filter, which contained two cartridge-shaped filter elements (Zeta Plus S, 3M—Itapetininga, SP, Brazil) composed of cellulose, cationic resin, and inorganic filter aids. The hot gelatin was recirculated for 5 min to ensure complete clarification. The filtered solution was collected in polypropylene bags and refrigerated overnight to induce gelatinization. Prior to concentration, the gel was melted in a 60 °C water bath and 6 L batches of molten gelatin were subsequently processed in a vacuum rotary evaporator (Heidolph, Schwabach, Germany) at 45 °C and 90 mbar. The evaporation continued until the soluble solids content reached roughly 5 °Brix. The concentrated gelatin was collected, placed in polypropylene bags, and stored under refrigeration until fully set. The resulting gelled product was cold-dried and fragmented using an industrial steel cutter—8 L—220 V (Skymsen, Brusque, SC, Brazil) before being stored in polypropylene bags.

#### 2.2.4. Physicochemical Characterization of Tambatinga Skin Gelatin

##### Yield

The extraction yield was determined by comparing the mass of dried gelatin obtained after processing with the initial dry weight of the cleaned fish skin. The result was expressed as a percentage on a dry weight basis, according to Equation (1):

(1) $$Yield=\frac{mass\;of\;dried\;gelatin}{mass\;of\;cleaned\;skin\;(d.b.)}\;\times \;100$$

In this equation, the *mass of dried gelatin* refers to the final granulated product collected after extraction, whereas the *mass of cleaned skin* represents the initial raw material weight, corrected to a dry basis.

##### pH

Three 6.67% (*w*/*v*) gelatin solutions were prepared in deionized water at 60 °C. The pH was measured using a calibrated digital potentiometer (Testo, CPQ, Brazil) and results expressed as means ± standard deviations.

##### Amino Acid Content

The amino acid profile of the gelatin samples was analyzed using high-performance liquid chromatography (HPLC) equipped with a reversed-phase column, following the methods proposed by White et al. [27] and Hagen et al. [28] using a high-performance liquid chromatography (HPLC) with a reversed-phase column. The concentration of tryptophan was quantified separately using the enzymatic–spectrophotometric technique described by Spies [29].

##### Gel Strength (Bloom)

Gel strength was assessed using 6.67% (*w*/*v*) gelatin gels prepared in glass containers (60 × 81.4 mm) [30]. The gelatin was first hydrated in water at ambient temperature for 4 h and subsequently dissolved in a 65 °C water bath for 15 min with gentle swirling to ensure complete solubilization. The resulting solution was cooled to room temperature and conditioned in a 10 ± 1 °C water bath for 18 h prior to analysis. Measurements were performed with a TA-XT Plus texture analyzer (Stable Micro Systems, Godalming, UK) using a cylindrical acrylic probe (P/0.5; diameter = 12.7 mm) at a speed of 1.0 mm s^−1^. Gel strength was expressed as Bloom value (mean ± standard deviation, n = 6).

##### Gel Melting Temperature

Thermal transitions were examined by differential scanning calorimetry (DSC TA2010, TA Instruments, New Castle, DE, USA). Approximately 0.01 g of dried gelatin was weighed into hermetically sealed aluminum pans, and deionized water was added with a micropipette to achieve a moisture content of approximately 70%. The hydrated samples were analyzed against an empty reference pan under a nitrogen flow (45 mL min^−1^). The temperature was increased from 0 °C to 60 °C at a heating rate of 10 °C min^−1^. The melting (gel–sol) temperature and enthalpy were determined from the peak temperature and the corresponding endothermic area, respectively.

#### 2.2.5. Production of Tambatinga Gelatin Films

Based on preliminary optimization tests, gelatin (2 g per 100 g of film-forming solution, FFS) was hydrated in distilled water at room temperature for 30 h. The hydrated mixture was then heated in a thermostatic water bath at 55 °C for 30 min to ensure complete dissolution. Glycerol was subsequently incorporated as a plasticizer at two concentrations: 10 g (TGF10) and 20 g (TGF20) per 100 g of gelatin. The resulting solutions were homogenized by magnetic stirring for 5 min and degassed in an ultrasonic bath for 10 min to eliminate air bubbles.

Each formulation (51 g) was poured into Petri dishes and dried in a ventilated oven at 30 °C for approximately 20 h. After drying, the films were peeled off and conditioned in desiccators containing NaBr (54% relative humidity, 25 °C) for a minimum of four days. Samples used for microscopy, thermal, and infrared analyses were additionally equilibrated over silica gel (0% relative humidity) for at least seven days.

#### 2.2.6. Characterization of Tambatinga Gelatin Films

##### Thickness and Moisture Content

Film thickness was measured at ten random points (3 replicates/formulation) using a digital micrometer (±0.001 mm; Mitutoyo, Tokyo, Japan), and the mean value was recorded. Moisture content was quantified gravimetrically by oven-drying the films at 105 °C for 24 h, following the procedure described by Sobral et al. [31].

##### Scanning Electron Microscopy (SEM)

The surface morphology of the gelatin films was observed using a TM3000 scanning electron microscope (Hitachi Ltd., Tokyo, Japan) operated at an accelerating voltage of 15 kV. Micrographs were captured from random areas of each sample. To examine the internal microstructure, the films were cryo-fractured after rapid freezing in liquid nitrogen.

##### Fourier-Transform Infrared Spectroscopy (FTIR)

Infrared spectra were recorded using a Spectrum One spectrophotometer (PerkinElmer, Waltham, MA, USA) equipped with a Universal Attenuated Total Reflectance (UATR) accessory, following the method of Bergo et al. [32]. Spectra were collected in the range of 650–4000 cm^−1^ at a resolution of 4 cm^−1^ and processed using FTIR Spectrum 5.3 software.

##### Differential Scanning Calorimetry (DSC)

Thermal transitions of the films were analyzed using a DSC TA2010 (TA Instruments, USA). Approximately 0.01 g of each sample, previously conditioned over silica gel for seven days, was sealed in hermetic aluminum pans. The analyses were performed under a nitrogen atmosphere (45 mL min^−1^) at a heating rate of 5 °C min^−1^ from −20 to 130 °C. Two consecutive heating scans were carried out, and prior to each, the DSC cell was quenched with liquid nitrogen.

##### Mechanical Properties

Uniaxial tensile tests were conducted using rectangular film strips (5 cm × 2.5 cm, n = 12/formulation). Measurements were performed at room temperature (22–24 °C) with a crosshead speed of 1 mm s^−1^ [33]. Tensile strength (TS, MPa) and elongation at break (EB, %) were determined from the stress–strain curves, while the elastic modulus (EM, MPa) was obtained from the slope of the initial linear region.

##### Water Vapor Permeability (WVP)

The permeability of the films to water vapor was determined according to ASTM E96-80 [34]. Circular film specimens (3 replicates/formulation) were sealed over aluminum test cells containing 40 g of silica gel (0% RH; vapor pressure = 0 Pa). The cells were placed in desiccators maintained at 25 °C with distilled water at the bottom (100% RH; vapor pressure = 3.1691 kPa). Weight gain was recorded at 24 h intervals for nine consecutive days. The water vapor permeability (WVP) was calculated using Equation (2) and expressed in g·mm·cm^−2^·h^−1^·kPa^−1^:

(2) $$WVP=\frac{∆G}{∆T}\left(\frac{\delta }{A.∆P}\right)$$

where ΔG/ΔT is the slope of the linear region of the mass gain vs. time curve (g·s^−1^), δ is the mean film thickness (m), A is the permeation area (32.17 m^2^), and ΔP is the vapor pressure difference across the film (3.1691 kPa).

##### Gloss

Gloss was evaluated at 20° and 60° incidence angles using a glossmeter (NGL 20/60, Rhopoint, Bexhill-on-Sea, East Sussex, UK). The measurements were carried out in triplicate, at 5 random points of each film, and results were expressed as means of gloss units (G.U.).

##### Color

Film color parameters were analyzed using a colorimeter (MSEZ 1049, HunterLab, Reston, VA, USA) with a D65 illuminant, 10° viewing angle, and 3 cm aperture, following the CIELab color system [35]. Each sample (n = 3) was placed over a standard white background, and the L*, a*, and b* values were recorded. The total color difference (ΔE*) was calculated using Equation (3):

(3) $${∆E}^{*}=\sqrt{\left[{\left(L^{*}-L_{0}^{*}\right)}^{2}+{\left(a^{*}-a_{0}^{*}\right)}^{2}{+\left(b-b_{0}^{*}\right)}^{2}\right]}$$

where $L_{0}^{*}$, $a_{0}^{*}$, and $b_{0}^{*}$ (94.01 ± 0.04, −0.76 ± 0.01, and 1.94 ± 0.03, respectively) correspond to the reference values of the standard white plate.

##### UV/Visible-Light Barrier

The optical transmittance of the films was measured using a UV–Vis spectrophotometer (Lambda 35, PerkinElmer, Waltham, MA, USA). Film strips (10 × 100 mm, 3 replicates/formulation) were mounted in the cuvette holder, and transmittance spectra were recorded from 200 to 800 nm in transmission mode [35].

### 2.3. Statistical Analysis

Statistical analysis of all collected data was performed using Analysis of Variance (ANOVA), followed by Tukey’s test to identify significant differences (*p* < 0.05). Data processing was carried out using the Minitab trial version program (Minitab 16.1.0, Minitab Inc., State College, PA, USA).

## 3. Results and Discussion

### 3.1. Tambatinga Skin and Gelatin Characterization

The physicochemical aspects of gelatin extracted from fish skin can be affected by the species, type of pretreatment (alkaline and/or acid), temperature, and processing time. For example, the approximate composition of Tambatinga skin showed higher protein (84.2%, dry matter) and lipid (13.1%, dry matter) content compared to other fish species (Table 1). For instance, the reported protein values for Red Tilapia (*Oreochromis nilótica*), Walking catfish (*Clarias batrachus*), and Striped catfish (*Pangasius sutchi fowler*) skins were 29.1%, 31.0%, and 33.7%, respectively [36]. Giacomelli da Silva et al. [37] observed that jundiá (*Rhamdia quelen*) skin have 89.3% of protein and 5.9% of lipids (Table 1). Additionally, when comparing protein content results, the nitrogen-to-protein conversion factor must be taken into account. According to the ASTM standard method [23] for determining protein in leather, the proper conversion factor for most different sources can vary between 5.44 and 5.8. Indeed, conversion factors of 5.4, 5.5, and 5.8 were used by Muyonga et al. [19], Giacomelli da Silva et al. [37], and Binsi et al. [38], respectively, in the determination of protein content in fish skins. Evaluating the specific conversion factors for different species from the Brazilian coast, Diniz et al. [24] found an average value of 5.71. All these values are within the range indicated by the ASTM standard, which recommends using the median value of 5.62.

The extraction process from Tambatinga skin resulted in a gelatin yield of approximately 35% (Table 2). Arnesen et al. [39] obtained a similar result producing Atlantic salmon (*Salmo salar*) skin gelatin. Considering the dry matter of skins, these authors found a yield value of 39.7% ± 2.2% slightly higher than the value determined in this work. Other studies showed lower yield results for other aquatic sources as by Alves et al. [40], which performed the gelatin production from salted and fresh cod Atlantic fish, found yield ranges from 6.7 to 18.5%. Two freshwater—Chinese longsnout catfish (*Leiocassis longirostris Günther*) and silver carp (*Hypophthalmichthys molitrix*)—and two marine fishes—salmon (*Oncorhynchus*) and Alaska pollack (*Theragra chalcogramma*)—were used by Yang et al. [41] for gelatin production. These authors found yield values of 21.8% ± 1.1%, 22.9% ± 1.1%, 23.2% ± 1.3%, and 21.5% ± 0.5%, respectively, lower than those observed in Table 2.

The relatively low yield value may be attributed to the elevated fat content of the Tambatinga skin (Table 1). Despite this, the filtration step proved effective, as evidenced by the low turbidity of the obtained gelatin (13.8 NFU). The final pH of the gelatin solution was 5.18 ± 0.16, and the gel strength reached 263.9 ± 5.3 Bloom, which classifies it as a high-Bloom gelatin. The highest value of gel strength corroborates what was reported in the literature on warm-water fishes [7]. Considering the classification of high (200–300), medium (100–200), and low-Bloom (50–100) [8], the obtained material fits into the first group. According to the standard analysis guide developed by the Gelatin Manufacturers Institute of America [30], the desirable characteristics for gelatin used as a hard capsule should be pH 4.5–5.5 and gel strength between 240 and 300 Bloom. Regarding the quantitative parameters of Tambatinga skin gelatin (Table 2), it could be considered for this application.

**Figure 1:**
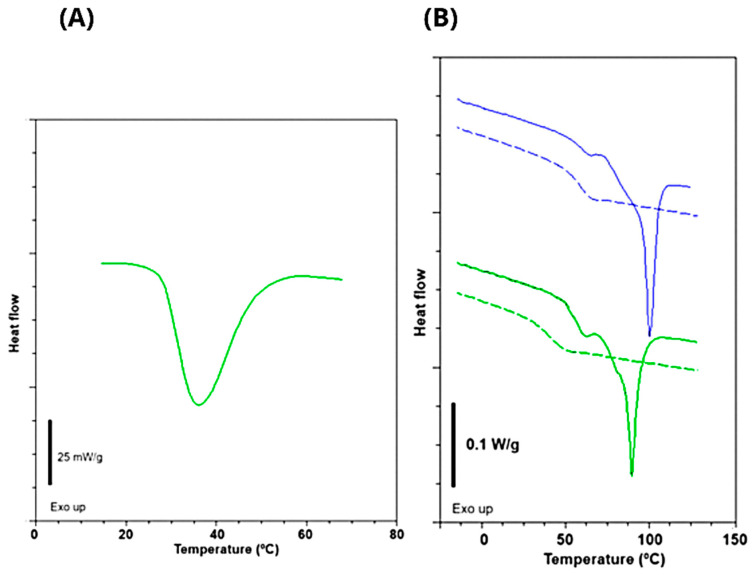
Thermograms of Tambatinga moist gelatin (**A**) and its films (**B**) with 10 (green) and 20% (blue) of glycerol: 1st scan (continuous line) and 2nd scan (dashed line).

The thermal curves of the moist gelatin samples presented only an endothermal event due to the gel melting (a gel-sol transition), typical of a physical gel (Figure 1A). The melting temperature and enthalpy of gelatin having 68.6 ± 0.2% (w.b) of moisture were 36.6 ± 0.6 °C and 7.0 ± 1.3 J/g, respectively (Table 2). Usually, gelatin derived from aquatic species has a low melting temperature [42]. This difference was due to the concentration of gelatin (~30%) in the analyzed samples. Determining the melting temperature under these conditions was important to ensure that the processing conditions for film production were at higher temperatures.

The protein material showed considerable content of the most characteristic amino acids Glycine (Gly), Proline (Pro), and Hydroxyproline (Hyp), constituting almost 46% of total amino acids (Table 3). Moreover, the content of Pro+Hyp was typical (~22% of total amino acids) of freshwater fish skin gelatin.

It was well established in the literature that gelatin’s physical and structural properties are related to the amino acid composition and molecular weight distribution. The presence of a higher content of proline (12.47 ± 0.05%) and hydroxyproline (9.84 ± 0.01%) (Table 3), when compared to other fish species [36,37,41], reinforces the best rheological behavior. The lower gel strength often reported for gelatin from aquatic species is believed to be due to the lower content of the Proline (Pro) and Hydroxyproline (Hyp) set when compared to mammalian sources [43]. Fan et al. [44] have highlighted the specific role of Hyp in the formation of intra- and intermolecular hydrogen bonds, which significantly enhances the structuring of the gelatin network. Nevertheless, a lower proportion of these amino acids (Pro+Hyp) may favor film deformation [45].

### 3.2. Tambatinga Gelatin Films Characterization

#### 3.2.1. Visual Aspect and Scanning Electron Microscopy (SEM)

**Figure 2:**
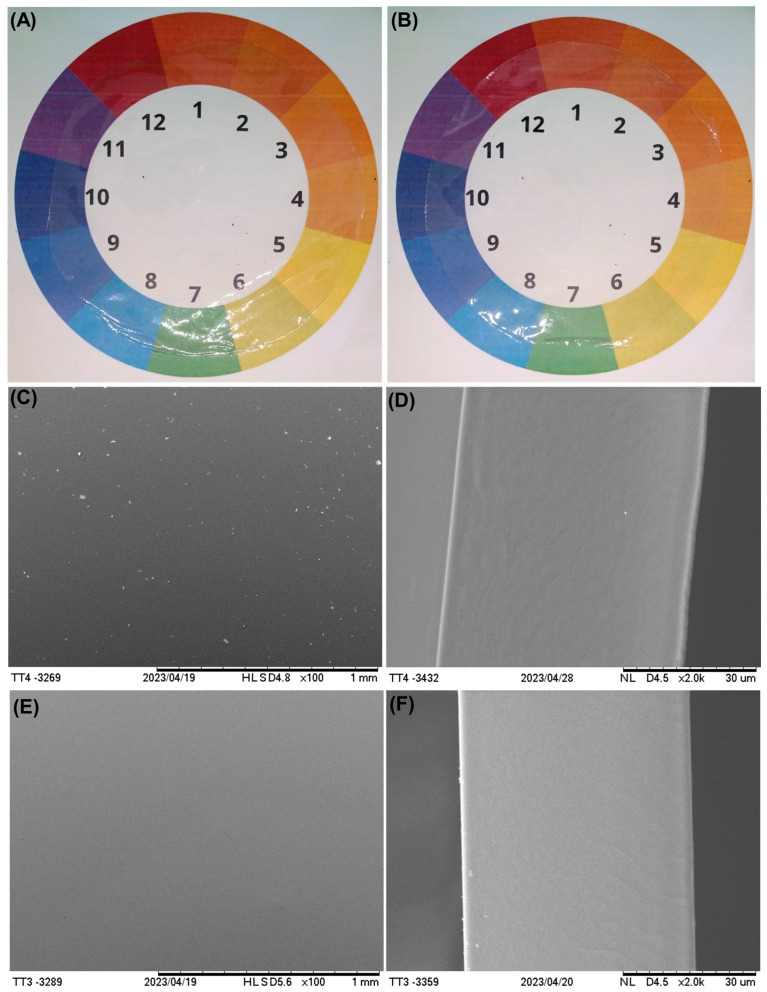
Visual aspect and scanning electron micrographs of air-side surface of Tambatinga skin gelatin films. (**A**,**B**) Visual aspect of films containing 10% and 20% glycerol, respectively. (**C**) Surface of film containing 10% glycerol (TG10); (**D**) Cryo-fractured cross-section of film containing 10% glycerol (TG10); (**E**) Surface of film containing 20% glycerol (TG20); (**F**) Cryo-fractured cross-section of film containing 20% glycerol (TG20).

Easy-to-handle, translucent, and high-gloss films with smooth and homogeneous surfaces were produced (Figure 2A,B). The transparency of these films is advantageous, as it allows clear visualization of the packaged product, which can attract consumers and enhance product acceptance. Moreover, being a biomaterial, these films offer a strong environmental appeal, combining biodegradability with sustainability, features increasingly valued by both industry and consumers, as previously demonstrated by Bonilla et al. [46].

SEM images showed that the surfaces of the films produced with 20% glycerol (Figure 2E) were uniform and smooth, whereas those made with 10% glycerol (Figure 2C) showed small particles. These imperfection points may be due to non-solubilized gelatin fragments or residual air bubbles present before drying. Despite characterizing possible breaking points, the effect of the imperfections noted in the formulation containing 10% glycerol (TGF10) was not observed on its mechanical properties. Moreover, no pores were observed in the internal structure of either formulation (Figure 2D,E). This result indicated that all components (gelatin, glycerol, and water) were well mixed and that any imperfections were only superficial. Liu et al. [47] also found a smooth, homogeneous structure when analyzing films produced from commercial cold-water fish gelatin.

#### 3.2.2. Fourier-Transform Infrared Spectroscopy (FTIR)

**Figure 3:**
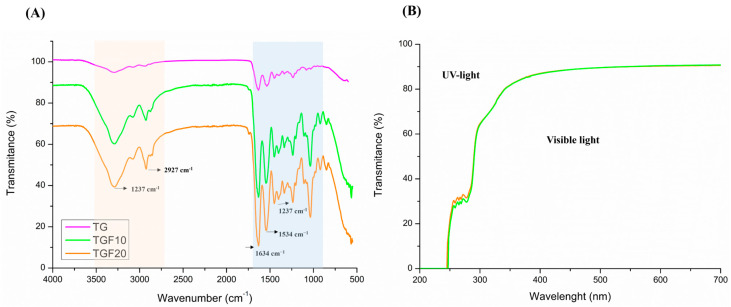
FTIR spectra (**A**) and transmittance scanning (**B**) in ultraviolet (400–200) and visible light (700–400 nm) of pure Tambatinga gelatin (pink) and Tambatinga gelatin films with 10% (green) and 20% (orange) and of glycerol. The highlighted areas in the FTIR spectrum indicate regions of difference between the peaks in the films and in pure gelatin.

The presence of a peak at 1035 cm^−1^ in the FTIR spectra of films (Figure 3A), absent for pure gelatin, may indicate an interaction between the protein and the plasticizer (glycerol) via hydrogen bonds. For films, the signal at 1237 cm^−1^ may be related to the splitting of amide III C–N and N–H bonds or even vibrations of glycerol groups [35,48]. The spectra also show bands at 1634 cm^−1^ (amide I) related to C=O stretching and 1534 cm^−1^ (amide II) representing N–H bending and C–N stretching (Figure 3). Other peaks between 2927 and 3291 cm^−1^ were observed only for films. As reported by Liu et al. [47], these bands are related to the amide-A and B regions, assigned to N–H and –OH stretching coupled with hydrogen bonds. Nevertheless, no effect of increasing the glycerol concentration was observed on the characteristic spectra.

#### 3.2.3. Thickness and Moisture Content

The main physical properties of Tambatinga gelatin films are present in Table 4. Glycerol concentration did not affect the thickness nor moisture content of the Tambatinga gelatin films (Table 4). Even doubling the glycerol content, which is highly hygroscopic, the results indicate similar water absorption during the conditioning period. Changes in the thickness can affect some physical aspects, especially the mechanical and barrier properties. Furthermore, the lack of difference in thickness and moisture between films produced with 10% (TGF10) and 20% plasticizer (TGF20) indicates good process control during film preparation, and conditioning. Other studies have observed the effect of the plasticizer on the physical characteristics and hygroscopicity of biopolymers-based films [49,50].

#### 3.2.4. Differential Scanning Calorimetry

The thermal curves of the Tambatinga skin gelatin films showed typical behavior for gelatin-based materials (Figure 1B). Following the first scan, an endothermic peak appears, characteristic of melting the crystalline fraction of the biopolymer (Figure 1B). This event corresponds to the helix-coil (sol–gel) transition and is typical of gelatin-based films [31,48]. In addition, it is confirmed the plasticizer effect of glycerol by reducing the T_g_ and T_m_ temperatures was also observed (Table 4). Melting temperatures (T_m_) and enthalpies (ΔH_m_) dropped from 100.3 °C and 23.8 J/g for films containing 10 g glycerol/100 g gelatin to 89.7 °C and 19.8 J/g for films containing 20 g glycerol/100 g gelatin (Table 4). According to the gel theory, a plasticizer acts by weakening the polymer-polymer interaction, resulting in a more flexible material [51]. Therefore, the increase in the plasticizer concentration generally reflect in lower thermal properties [32,52,53]. During heating, the crystalline structures were destroyed, and after cryogenic cooling, new junctions were not formed, giving rise to a purely amorphous material [31]. The absence of the sol–gel transition event in the second scan is typical behavior of gelatins from different origins.

The glass transition phenomenon describes the mobility of molecules in the amorphous phase, passing from a brittle state to a highly viscous form [54]. Apparently, glycerol has a protective effect against the helix-coil transition, reducing crystalline nuclei [31,55]. The films containing 10 g of glycerol/100 g of gelatin (TGF10) shown Tg1 and Tg2 at 60.1 °C and 80.7 °C, while the films produced with 20 g of glycerol/100 g of gelatin (TGF20) show 58.3 °C (Tg1) and 77.9 °C (Tg2) (Table 4). Generally, at this temperature range, Tg is associated with the plasticized gelatin-rich phase, and the presence of two close events may be related to clusters of different molecular weights [31,56].

Depending on process conditions and gelatin characteristics, especially the amino acid content, different thermal behavior for fish gelatin-based materials can be found in the literature [37,56,57]. Usually, an enhancement in thermal events is associated with a higher proline and hydroxyproline content. Lower levels of these amino acids reduce gelatin’s ability to restructure into a triple helix during gelation [58,59]. Indeed, as previously discussed, Tambatinga gelatin presented more of these amino acids than other fish species [37,41,42]. The T_g_ and T_m_ values for films produced from Tambatinga gelatin were higher than other fish gelatin-based films [57,58,60]. Tongnuanchan et al. [57] reported control films made from 3.5% gelatin extracted from Tilapia skin and 30% (protein content) of glycerol, with T_g_, T_m_, and enthalpy values of 41.30 °C, 76.92 °C, and 13.56 J/g, respectively. Kchaou et al. [60] studied commercial fish gelatin (type A) based films which presented a glass transition temperature of 50.9 °C.

Through the DSC results, it is also possible to measure the crystallinity of the gelatin films. According to Arvanitoyannis et al. [55], during the gelatin gelation and at a molecular level, the renaturation of polypeptide chains into triple helix structures occurs as in native collagen. Thus, by relating the enthalpy measured in the thermal curves of the films with the enthalpy of the native collagen, we obtain the gelatin’s renaturation degree. Considering the value found in the literature [31,55] for collagen enthalpy (62.05 J/g), we found crystallinity values of 31.9 and 38.37% for TGF20 and TGF10, respectively. The action of glycerol against the sol–gel transition, reducing the junction of helix-coil structures, can explains this result. In the study by Sobral et al. [31], the increase in plasticizer concentration (above 25 g of sorbitol/100 g of gelatin) also caused a reduction in the crystallinity of pigskin gelatin films.

#### 3.2.5. Mechanical Properties

Mechanical properties were affected by the amount of plasticizer (Table 4). The increasing glycerol concentration caused a reduction in tensile strength and increasing in the elongation at break (Table 4). Films with 20% of the plasticizer (TGF20) showed a tensile strength (TS) and a maximum elongation at break of 37.9 ± 3.3 MPa and 159.1 ± 3.2%, respectively, while the sample produced with 10% of glycerol (TGF10) presented 59.4 ± 5.8 MPa and 116.0 ± 3.6%, respectively (Table 4). Furthermore, the less glycerol content (TGF10) reflected in a higher elastic modulus (13.8 ± 1.9 MPa). This phenomenon was a consequence of the Tg variation due to the plasticizing action of glycerol, which reduces intermolecular interactions and confers greater mobility to the protein network [31]. Similar behavior was observed by Staroszczyk et al. [61] producing Salmon fish gelatin (1 g/100 mL of water) films with different glycerol concentrations (15 and 20% related to protein content). The authors observed that the increase in plasticizer content resulted in a lower tensile strength (35.6 to 7.1 MPa) and higher elongation at break (7.2 to 49.8%).

The favorable mechanical properties founded for Tambatinga skin gelatin films (Table 4) when compared to other gelatin-based films reported in the literature [35,48,61] can be attributed to the high amino acid content (Table 3). For instance, Morais et al. [62] found TS and elasticity values of 2.68 ± 0.54 MPa and 27.48 ± 5.21%, respectively, for their Nile-Tilapia gelatin films (formulated with 4.43 g of gelatin and 3.40 g of combined plasticizers glycerin/pectin). The origin (mammal or aquatic) and gel strength of the raw material, in addition to the protein/plasticizer ratio, can alter the mechanical behavior of the films. Bonilla and Sobral [35] used pigskin gelatin (260 Bloom) at a concentration of 4 g/100 mL of distilled water with 0.2% of glycerol to produce control films. These authors found tensile strength, elongation at break, and elastic modulus of 2.4 MPa, 4.4%, and 95 MPa, respectively. Tessaro et al. [48] evaluated the mechanical properties of control films produced by 4 g of bovine gelatin (225 Bloom)/100 g of distilled water and glycerol (25 g/100 g of protein) and observed that film presented tensile strength of 27.2 ± 1.7 MPa, elongation at break of 14.5 ± 0%, and elastic modulus of 8.5 ± 0.8 MPa.

#### 3.2.6. Water Vapor Permeability (WVP)

The water vapor permeability (WVP) values did not show a statistically significant difference (*p* > 0.05) across the glycerol content levels tested (Table 4). Furthermore, the WVP values determined for the Tambatinga gelatin films were lower than those previously documented for films made from silver carp [63] and food grade commercial fish gelatin [64] films.

The matrix composition, type of protein-plasticizer interaction, thickness, relative humidity gradient, and eventually porosity of the films can influence water vapor migration across the biopolymer matrix. Typically, protein-based films are not good water vapor barriers due to the presence of polar groups in the film matrix. Therefore, many authors evaluated the addition of other biopolymers and extracts to improve this property [61,65]. The outcomes have been mixed, largely dependent on the specific formulation. Although it was not the objective of this work, studying the combination of Tambatinga skin gelatin with other biopolymers may further improve its physicochemical aspects.

For instance, the addition of passion fruit extract to edible food wraps derived from tilapia bone gelatin resulted in a counterintuitive decrease in water vapor permeability (WVP), despite the extract’s inherent hydrophilic nature [66]. The authors proposed that the hydrophilic groups facilitated the formation of a more continuous and homogeneous film-forming matrix. A consistent, defect-free matrix effectively creates a more challenging pathway for water molecule transmission. This same principle may account for the lack of a substantial WVP change following the increase in glycerol in our Tambatinga skin gelatin films. Even though glycerol is hygroscopic, a higher amount of the plasticizer may have made the film-forming matrix more closely packed and cohesive, ultimately maintaining its barrier properties against water diffusion.

#### 3.2.7. Color and Gloss Properties

Tambatinga gelatin films with 20 and 10% of glycerol (TGF20 and TGF10, respectively) showed no significant difference regarding color parameters (Table 4). TGF20 and TGF10 showed high luminosity (89.9 ± 0.2 and 90.2 ± 0.1, respectively), low green–redness a* (−1.1 ± 0.0), low blue–yellowness b* (2.7 ± 0.2 and 2.8 ± 0.3, respectively), and very low color difference values (3.8 ± 0.2 and 3.6 ± 0.2, respectively), meaning that these materials were almost colorless. Furthermore, the films presented very low opacity (Opacity → 0) because they were very translucent.

Color and gloss are important parameters in the acceptability of food products. Considering film packaging, they must be visually attractive and functional, as they can directly influence consumer choice. Factors such as species, skin pigmentation, extraction temperature, and Maillard reaction products can change the color of gelatins [36,67], affecting the film’s appearance. Additionally, film gloss indicates the degree of surface polish and can affect a product’s attractiveness. Tambatinga gelatin films with 20 and 10% glycerol showed gloss of 77.1 ± 14.6 a and 81.4 ± 5.7, respectively, when evaluated at 20° (Table 4). At 60°, the values were 145.3 ± 3.3 and 152.5 ± 2.2 for TGF20 and TGF10, respectively. This result indicates that no insoluble particles or aggregates that could avoid light transmission were present in this material, and that the surface was sufficiently smooth.

#### 3.2.8. UV-Vis Light Barrier

The UV-vis light transmittance curves of gelatin films from Tambatinga containing 20 (TGF20) and 10% glycerol (TGF10) were similar (Figure 3). Both showed transmittance close to zero in the UV region between 200 and 250 nm. In the 250–300 nm region, the films have low transmittance, around 30%.

The effective UV barrier performance below 300 nm is primarily due to the presence of specific aromatic and heterocyclic amino acids inherent to the gelatin structure (Figure 3B). According to Belitz et al. [68], phenylalanine, tyrosine, and tryptophan absorb light in the range of 200–230 nm and 250–290 nm. Furthermore, histidine, cysteine, and methionine typically exhibit absorption peaks in the 200 and 210 nm range. The identification of most of these compounds in Tambatinga skins (Table 3) indicates the films possess a robust capacity to block UV radiation. Consequently, these films present potential for protecting food products containing easily oxidized or photosensitive components. This observed barrier behavior is consistent with results found in the literature studies that assessed gelatin films from different origins [35,47,48,63].

In the visible spectrum, the films demonstrated high transmittance (over 80%) which is supported by their minimal opacity values (Figure 3B). Notably, the opacity values for the Tambatinga gelatin films tended toward zero (Table 4), performing better in this regard than most formulations reported by Morais et al. [65] for tilapia gelatin films, which displayed opacity greater than 1%.

## 4. Conclusions

Gelatin was produced from Tambatinga fish skin, an abundant co-product of the Brazilian aquaculture industry, and subsequently utilized in the fabrication of biopolymer films. This approach directly supports the principles of the circular economy by transforming fish processing co-products into a high-value material, thereby minimizing waste and promoting sustainable development within the food sector.

The extraction process yielded a good efficiency (35.5%) and was effective in removing pigments and fats, resulting in gelatin with low turbidity (13.8 ± 0.3 NFU). The high concentration of proline (12.47%) and hydroxyproline (9.47%) explains the strong gel strength (263.9 Bloom), aligning with the robust technological characteristics typically observed in gelatin extracted from warm-water fish species.

The resulting Tambatinga gelatin/glycerol films were transparent, possessing a continuous, smooth, and high-gloss surface. While glycerol concentration did not significantly impact film thickness or moisture content, increased plasticizer content reduced the tensile strength, glass transition, and melting temperatures. Overall, the films demonstrated favorable functional properties, including strong barrier capacity in the UV region (below 300 nm), indicating their potential utility for packaging photosensitive compounds and contributing to the development of sustainable food packaging solutions.

## Figures and Tables

**Table 1:** Proximate composition of Tambatinga skin compared with literature data for other species.

Fish Species (Skin)	Moisture (%)	Protein (%) *	Lipids (%) *	Ash (%) *	Reference
Tambatinga	69.85	84.2 ± 1.4	13.1 ± 0.7	0.7 ± 0.1	
Jundiá	70.6 ± 0.8	89.3 ± 2.9	5.9 ± 0.4	3.4 ± 0.3	[37]
Red Tilapia	70.43 ± 0.2	29.07 ± 0.31	-	0.51 ± 0.09	[36]
Walking catfish	62.47 ± 0.34	31.01 ± 0.48	-	0.52 ± 0.23	[6]
Bigeye snapper	52.79 ± 0.53	25.19 ± 0.85	1.2 ± 0.06	20.2 ± 0.64	[38]

Values expressed as mean ± standard deviation. * Determined in dry matter.

**Table 2:** Physicochemical characteristics of Tambatinga skin gelatin.

Properties	Tambatinga Skin Gelatin
Yield (%)	35.5 ± 3.5
Turbidity (NFU)	13.8 ± 0.3
pH	5.18 ± 0.16
Gel strength (Bloom)	263.9 ± 5.3
Melting temperature (°C)	36.6 ± 0.6
Melting enthalpy (J/g)	7.0 ± 1.3

Values expressed as mean ± standard deviation.

**Table 3:** Amino acid composition of Tambatinga skin gelatin.

Amino Acid	g/100 g of Total Amino Acids
Aspartic acid	5.39 ± 0.04
Glutamic acid	9.74 ± 0.03
Serine	3.23 ± 0.01
Glycine	23.44 ± 0.09
Histidine *	0.67 ± 0.03
Arginine	8.70 ± 0.13
Threonine *	2.34 ± 0.07
Alanine	9.76 ± 0.03
Proline	12.47 ± 0.05
Tyrosine	0.41 ± 0.02
Valine *	2.12 ± 0.04
Methionine *	1.10 ± 0.08
Cysteine	0.47 ± 0.02
Isoleucine *	1.25 ± 0.02
Leucine *	2.62 ± 0.01
Phenylalanine *	1.80 ± 0.03
Lysine *	3.57 ± 0.02
Hydroxyproline	9.84 ± 0.01
Tryptophan *	ND < 0.05

ND: not detected (˂0.05). Values expressed as mean ± standard deviation. * essential amino acids.

**Table 4:** Physical parameters of Tambatinga gelatin films.

Properties	TGF20	TGF10
Thickness (µm)	72.2 ± 8.0 ^a^	70.4 ± 7.1 ^a^
Moisture (%)	11.6 ± 0.0 ^a^	11.1 ± 0.7 ^a^
T_g1_ (°C) 1st scan	58.3	60.1
T_g2_ (°C) 1st scan	77.9	80.7
T_m_ (°C) 1st scan	89.7	100.3
ΔH_m_ (J/g) 1st scan	19.8	23.8
T_g_ (°C) 2nd scan	41.5	59.7
Tensile strength (MPa)	37.9 ± 3.3 ^b^	59.4 ± 5.8 ^a^
Elongation at break (%)	159.1 ± 3.2 ^a^	116.0 ± 3.6 ^b^
Elastic modulus (MPa)	4.9 ± 0.7 ^b^	13.8 ± 1.9 ^a^
Water vapor permeability (g·mm/cm^2^·h·kPa)	0.028 ± 0.00 ^a^	0.025 ± 0.00 ^b^
Gloss units (G.U.) at 20°	77.1 ± 14.6 ^a^	81.4 ± 5.7 ^a^
Gloss units (G.U.) at 60°	145.3 ± 3.3 ^B^	152.5 ± 2.2 ^A^
L*	89.9 ± 0.2 ^a^	90.2 ± 0.1 ^a^
a*	−1.1 ± 0.0 ^a^	−1.1 ± 0.0 ^a^
b*	2.7 ± 0.2 ^a^	2.8 ± 0.3 ^a^
ΔE*	3.8 ± 0.2 ^a^	3.6 ± 0.2 ^a^
Opacity (%)	0.4 ± 0.1 ^a^	0.6 ± 0.2 ^a^

TGF20: Tambatinga gelatin films with 20% of glycerol; TGF10: Tambatinga gelatin films with 10% of glycerol. Different letters in the same row represent a statistically significant difference (*p* ≤ 0.05).

## Data Availability

The original contributions presented in this study are included in the article. Further inquiries can be directed to the corresponding author.
